# Introducing AHA's New President: Joseph C. Wu, MD, PhD, FAHA

**DOI:** 10.1161/JAHA.123.031618

**Published:** 2023-07-25

**Authors:** Bridget M. Kuehn

As a young person, Joseph C. Wu, MD, PhD, the Simon Stertzer, MD, Professor of Medicine and Radiology and Director of Stanford Cardiovascular Institute, was drawn to cardiology because he was fascinated by the beating heart—“the center of melodic, rhythmic motion that makes it the most palpable organ of the human body.” Now, Joe (as he is known to colleagues and friends) regularly observes the behavior of beating cardiomyocytes grown from patients' cells in his laboratory at the Biomedical Innovation building across from the Stanford University Hospital.

“We take patient's blood cells, convert them into induced pluripotent stem cells and differentiate them into heart cells in a dish,” he said. “We can also grow human engineered heart tissues in a dish.”

His work on human induced pluripotent stem cells (iPSCs) is on the cusp of revolutionizing cardiovascular drug studies. It is also bringing personalized medicine into reach for patients with genetic forms of heart disease. As he continues his ambitious research agenda, Dr Wu hopes to share his passion for cardiovascular health and science with the public, policymakers, and the next generation of cardiovascular researchers as he steps into the role of the 2023–2024 president of the American Heart Association.

“I want to raise public awareness about the importance of cardiovascular health because heart disease is the number one cause of mortality,” he said during an interview. “I also want to emphasize the importance of investing in biomedical research that will benefit our patients in the future and training the next generation of scientific researchers who will do this critical work for decades to come.”

## REACHING FOR THE STARS: AN AMERICAN TALE

Dr Wu lived with his family in Kaohsiung, Taiwan, until he was 9 years old. Tensions between China and Taiwan were simmering, and many families fled, fearing armed conflict. He and his family traveled from Taiwan to South America before eventually settling in Los Angeles, California, in 1981.

Dr Wu's father was a farmer and grew pears and apples in Central Valley, California, and Dr Wu helped his dad on the weekends.^1^ The demanding labor and unpredictability of weather and managing the farm taught him many lessons that would be helpful in running a laboratory years later. Looking up at the starry night in rural California, Dr Wu dreamed of becoming an astronaut or a pilot one day.

“I've always had a fascination with sky and space,” he said.

His dream was dashed, however, when he eventually required wearing glasses to correct his vision, which was disqualifying for a prospective pilot or astronaut at the time. By high school, he had shifted his gaze to being a scientist and eventually a physician. Dr Wu earned his bachelor's degree in biology from the University of California, Los Angeles (UCLA) in 1993 and completed his medical degree at Yale School of Medicine in New Haven, Connecticut, in 1997. He moved back to California to complete his doctorate degree (PhD) in molecular and medical pharmacology at UCLA in 2004.

**Figure 1:**
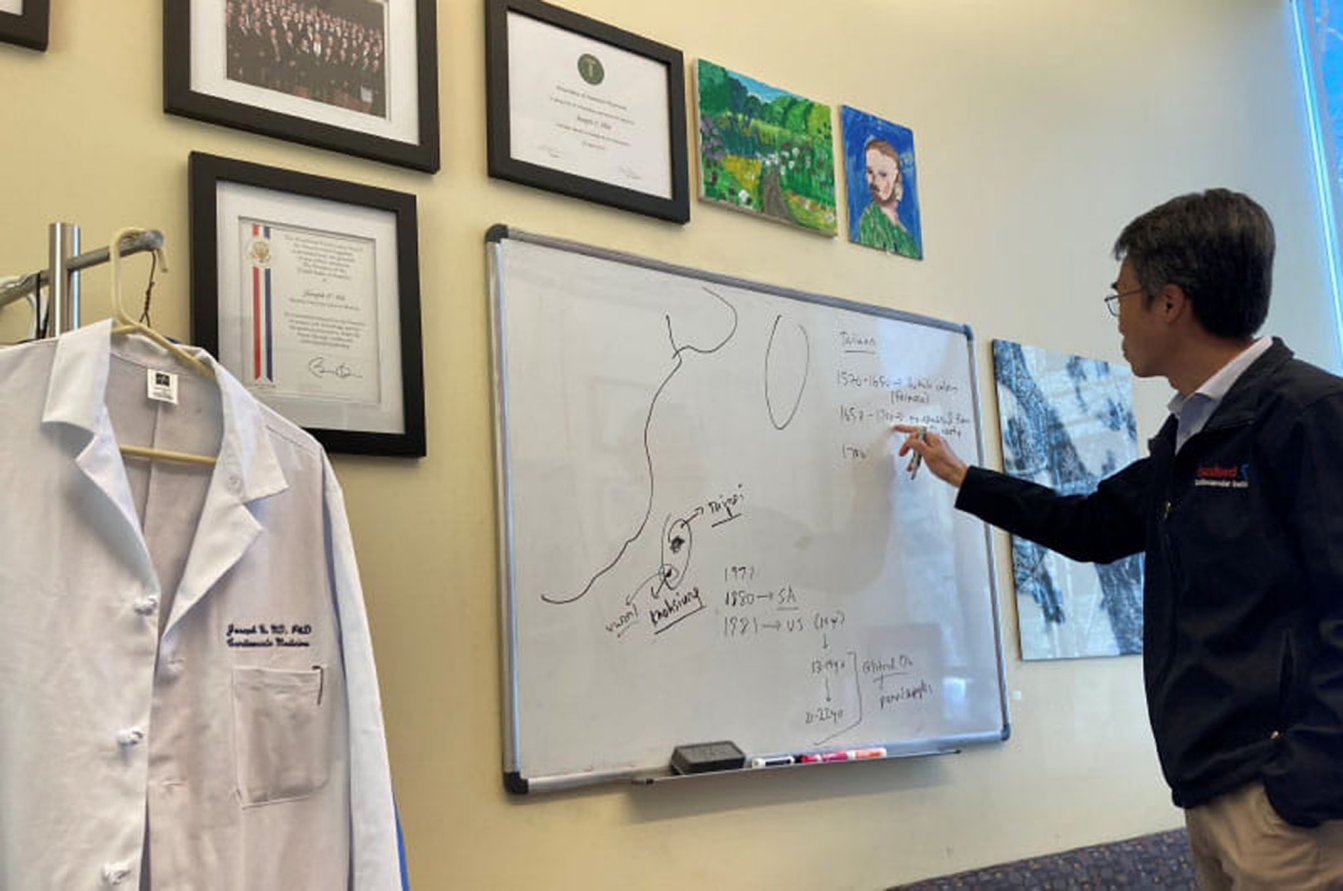
Dr. Joseph Wu arrived at Stanford University in 2004. His academic journey has led him to cutting‐edge research on induced pluripotent stem cells—blood or skin cells that have been reprogrammed so they can become any type of human cell. Reproduced with permission from American Heart Association.

Working with his mentor, the late Sanjiv Sam Gambhir, MD, PhD, who was developing revolutionary molecular imaging techniques to study gene expression in living animals and humans, was pivotal to his career, Dr Wu said. Dr Gambhir's focus was cancer, but Dr Wu wanted to adapt the innovative techniques to cardiology.^1^ Dr Gambhir became the head of the division of nuclear medicine at Stanford in 2003. He recruited Dr Wu to Stanford in 2004 with a joint appointment in radiology and cardiology, a laboratory space, start‐up funding, and protected research time (Figure 1).

“Sam had a tremendous influence on me,” Dr Wu said. “He was an innovative thinker and a true visionary. My PhD training in his lab, as well as his guidance when I was a junior investigator, provided me with a solid foundation as a physician scientist. Because of that, I've been able to work on areas that are quite cutting‐edge and translational.”

Dr Wu's dreams came full circle in 2016 when the National Aeronautics and Space Administration (NASA) selected him to work on a project to make space travel safer for humans, with a special focus on cardiac health.

“People will be traveling in space in the future,” he said. “It's not science fiction anymore.”

Dr Wu predicts space travel will become accessible in his grandchildren's lifetime. But first, scientists must work out a few key problems. They include the harmful effects of reduced gravity on the body, exposure to cosmic radiation, and learning how to induce chronic hibernation to allow people to sleep through their long journeys. Dr Wu's project focuses on understanding and preventing the effects of reduced gravity on human cells.

“When you are on earth, gravity helps strengthen your bones and muscles,” he explained.

He and his team sent 2‐dimensional iPSC‐derived cardiomyocytes to space, kept some on Earth, and worked with astronauts to compare them. They discovered that the cardiomyocytes in space had altered metabolism and contractability.^2^ In 2020, they sent 3‐dimensional (3D) iPSC‐derived engineered heart tissues into space and saw the same changes. This year, they sent 3800 iPSC‐derived cardiac organoids into space. Half of the 3D organoids were treated with medication to mitigate the metabolic and contractile changes; half were not. They are hoping to learn if the treatment strategy is effective.

## PATIENT‐CENTRIC RESEARCH

Dr Wu's NASA study is just 1 example of the innovative ways he is deploying iPSCs in combination with artificial intelligence, machine learning, next generation sequencing, genome editing, molecular imaging, and other cutting‐edge techniques to study cardiovascular health. For example, he has used patient‐specific and disease‐specific iPSCs to tease apart the mechanisms of inherited dilated cardiomyopathies,^3^ hypertrophic cardiomyopathy,^4^ long‐QT syndrome,^5^ congenital heart diseases,^6^ and chemotherapy‐induced cardiotoxicity.^7^, ^8^ Recently, he has been using iPSCs to model the effects of environmental pollution as well as addictive substances such as marijuana^9^ and vaping.^10^

“Using iPSCs, we can model many different kinds of diseases and understand how they affect patients and hopefully how to treat them,” he said.

For example, he has used this approach to study a large family with a cardiomyopathy‐causing mutation in *LMNA*.^11^ This gene encodes the protein lamin A. A total of 8 family members with the mutation had died of sudden cardiac death, he said.

“We worked out the mechanism of how the *LMNA* mutation causes cardiomyopathy and sudden cardiac death in this family,” Dr Wu said. They also discovered that the family members were prone to early‐onset hypertension, and they published a follow‐up study demonstrating how the mutation causes vascular endothelial dysfunction.^12^ Then, they screened for drugs that might counteract the disease mechanism and found treating the patients—who did not have the typical warning sign of high cholesterol—with the drug lovastatin could mitigate the vascular dysfunction.

“You work out the whole disease mechanism using iPSCs, you do genome editing to confirm, then you screen for drugs and find what works and what doesn't, and finally you bring the patient back for treatment,” he said. “You complete the whole cycle of from precision medicine to clinical trial in a dish concept, and all of this could be accomplished within about 1 year.”

Recently, Dr Wu showed how a genetic variation in the aldehyde dehydrogenase 2 (*ALDH2)* gene contributes to alcohol‐induced blood vessel harm and identified an existing drug that reduces the harm.^13^ Approximately 35% of people of East Asian descent, including Dr Wu, and >600 million people or about 8% of the global population have a genetic variation that causes facial flushing, headaches, and a rapid heartbeat when they consume alcohol. The genetic variation is also associated with an increased risk of coronary artery disease.^14^ These individuals produce a less effective version of the ALDH2 enzyme, which helps break down alcohol into a harmless by‐product, Dr Wu said.

“Anytime you drink alcohol, you have a build‐up of acetaldehyde, which precipitates the alcohol‐induced flushing,” Dr Wu explained. “That means anytime you drink, the acetaldehyde can cause DNA damage and oxidative stress in your body. The ALDH2 enzyme breaks down acetaldehyde to acetic acid, which is nontoxic. If you have a mutation that inactivates the ALDH2 enzyme, like I do, the vascular damage lasts much longer.”

By turning iPSCs from people with the variation into endothelial cells and exposing them to alcohol in the laboratory, Dr Wu and his team discovered that alcohol impairs the ability of the endothelial cells to relax. Using CRISPR/Cas9 gene editing, they corrected the *ALDH2* genetic variant and resolved the issue. They also showed that the *ALDH2* genetic variant increases oxidative stress and inflammation and decreases the production of the blood vessel dilator nitric oxide. Being exposed to alcohol further exacerbates these deficits, he explained.

Next, they screened for existing drugs that might counteract these effects. They discovered that the diabetes drug empagliflozin was a good prospect. In mouse studies, the drug improved vascular function, circulation, and endothelial cell health. In human iPSC studies, they showed that the drug worked by reducing oxidative damage and inflammation and increasing nitric oxide production. Dr Wu said this study is typical of the bench‐to‐bedside research that his laboratory does.

“Joe's discoveries have significantly pushed forward translational applications of patient‐specific iPSCs in cardiovascular medicine,” said Mingtao Zhao, PhD, assistant professor at the Center for Cardiovascular Research at Nationwide Children's Hospital and The Ohio State University College of Medicine in Columbus, Ohio, who is a former mentee of Dr Wu.

## STATIN SELECTION?

In addition to helping explain the causes of more rare conditions that harm heart health, Dr Wu and his colleagues have also solved some central mysteries in cardiology.

As 1 of the >40 million statin users in the country, Dr Wu has wondered about the drugs' effects throughout the body. Statins inhibit the production of low‐density lipoprotein cholesterol in the liver, but they also seem to help reduce inflammation, improve blood vessel function, and help prevent vessel blockages.^15^

“We showed that in iPSCs from patients with vascular dysfunction, the drug simvastatin can be used to improve vascular function,” he said.

When they began to interrogate the molecular mechanism, they discovered that simvastatin prevents epigenetic changes leading to the expression of genes that cause the endothelial cells to transition into stiffer, less functional mesenchymal cells.^16^ They found that they could inhibit this harmful change by blocking other steps in the pathway, which might lead to new strategies to treat vascular dysfunction.

In another study, Dr Wu and his colleagues compared the effects of 4 common statins—atorvastatin, lovastatin, simvastatin, and fluvastatin—on iPSC‐derived cardiomyocytes grown from 4 patients.^17^ They showed that each drug had distinct effects on gene expression on these cardiomyocytes. They also detected differences in the responses of cells from different individuals to the same drug.

“Not all statins are the same, and not all of us will respond to the same statin the same way,” he said.

That opens the possibility of testing drug effects in patients' iPSCs grown into different cell types—such as brain cells, heart cells, blood vessel cells, and muscle cells—to determine which would be most beneficial, Dr Wu explained, but he acknowledged that the time and costs of this individualized approach remain a barrier to implementation.

“The cost needs to come down dramatically, which is not possible at this moment but likely in the future,” Dr Wu said.

## ACCELERATING DRUG DISCOVERY

The more immediate impacts of Dr Wu's work may come from using the technology on a larger scale to facilitate drug safety research and drug testing.

“On a small scale, you could use this for precision medicine,” he explained. “On a large scale, you use it for clinical trials in a dish.”

He explained that, in the future, it would be possible to first use artificial intelligence or machine‐learning software to identify a prospective drug target for a condition. Then, investigators would test the therapy on conventional in vitro and in vivo mouse studies. Afterward, they would then use iPSCs from ≈1000 patients to confirm whether these drugs can block or activate the molecular target of interest. If it does, they would have more confidence to proceed to larger scale human studies.

“We are hoping to use this novel concept to accelerate the drug discovery process,” he said.

He explained that, traditionally, scientists test prospective drug candidates in mice models of the disease before moving on to human studies. However, many drugs that show promise in mice models fail in human trials. The use of mice lacking genetic diversity as well as fundamental differences between humans and mice may explain why so many drugs fail at this step, he explained.

“Drugs that work in mice may not work in humans, and vice versa,” he said. In addition, he noted that sometimes when scientists try to create a mouse model by inducing a mutation that causes the disease in humans, the mouse does not manifest the disease. Lastly, he commented that “even if a drug works beautifully well in 100 inbred mice, you're assuming that it will work equally well for 100 genetically diverse humans, and almost certainly that won't be the case in the real world.”

Using iPSCs from large numbers of patients first could help reduce the failure rate and provide insights into why some patients respond and some do not. It also gives drug companies a way to screen multiple candidate therapies more quickly with great cost reduction to identify the most promising before proceeding to laborious human clinical trials. He mentioned that Congress has recently passed the FDA Modernization Act 2.0, which emphasizes similar ideas.^18^

“In many applications, it's much more relevant and efficient because you are screening for drugs using human samples with the actual disease of interest,” he said. Using this approach could shave a considerable amount of time from the 10 to 15 years it typically takes to bring a new drug to market, and it could also help reduce the costs of drug development by reducing the high failure rate, he said.

## “WORK HARD, WORK SMART, WORK TOGETHER”

During his prolific career, Dr Wu has published >500 manuscripts and has been listed as one of the top 0.1% of highly cited researchers by the Web of Science for the past 5 years. It is all part of his commitment to disseminate his knowledge and tools as widely as possible. His laboratory has shipped out >3700 vials of iPSCs to >500 laboratories in the United States and abroad.

“We are trying to ramp up our approach, expand more, and teach other people to do it,” he said. “The more people use it; the faster science and medicine will advance.”

**Figure 2:**
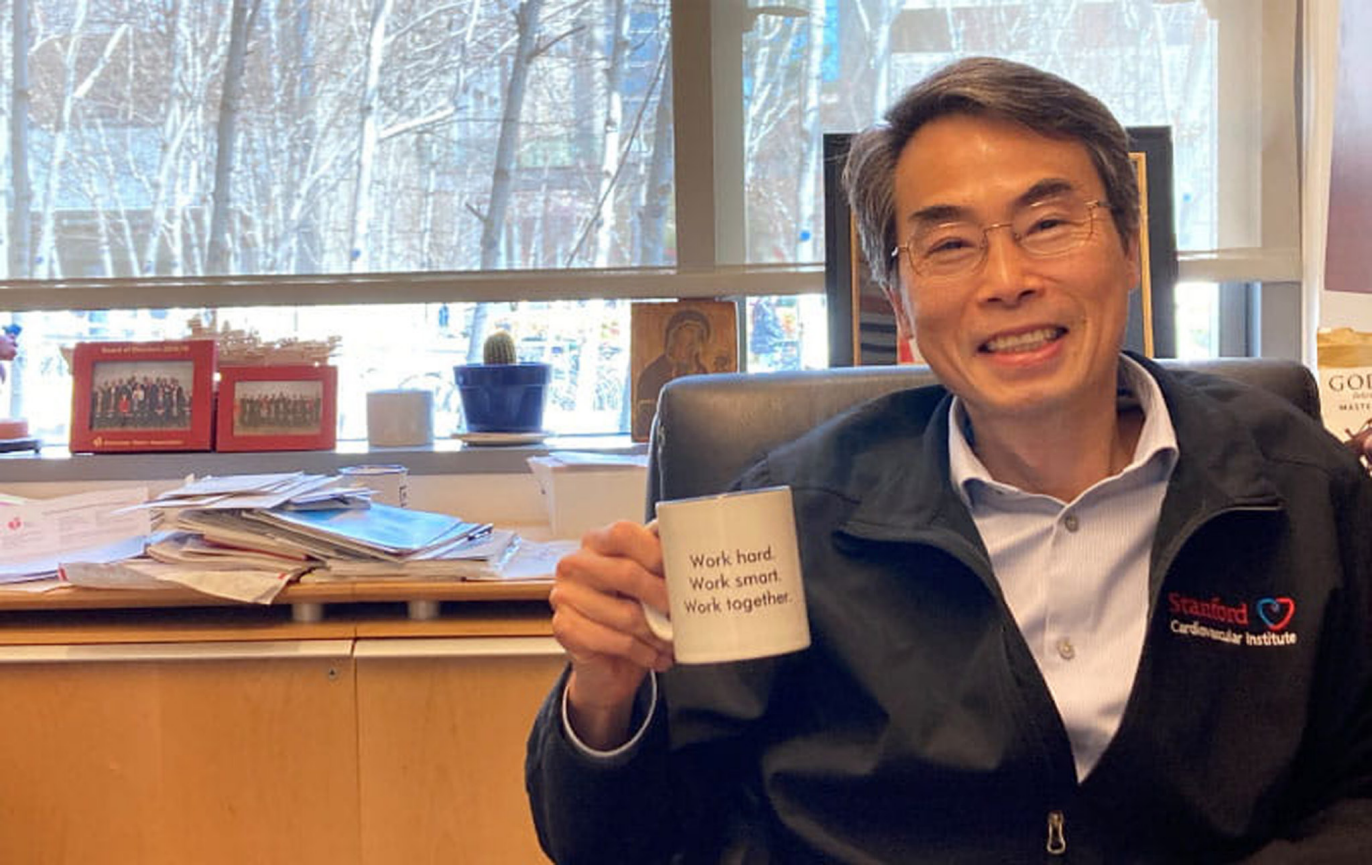
In his office, Dr Joseph Wu's coffee mug displays the mantra he teaches his mentees: “Work hard/Work smart/Work together.” The mug was a gift from one of those mentees. Reproduced with permission from American Heart Association.

To date, 48 of his trainees are principal investigators of clinical or research studies in the United States or abroad. His motto “Work Hard, Work Smart, Work Together” created a productive and rewarding environment to work in, according to former mentees (Figure 2).

“Being a trainee in the Wu lab has had a profound personal impact on me,” said Mingxia Gu, MD, PhD, assistant professor at the Center for Stem Cell & and Organoid Medicine at Cincinnati Children's Hospital Medical Center in Ohio. She continues to apply Dr Wu's motto in her own work. “[It] emphasizes the importance of dedication, strategic thinking, and fostering a collaborative environment to achieve successful outcomes.”

Karim Sallam, MD, an assistant professor of medicine at Stanford University, said it was initially intimidating working in such a fast‐paced environment with so many other accomplished trainees, but Dr Wu's emphasis on teamwork created a supportive environment where laboratory members used their expertise to help each other.

“No one is good at everything, but a team can be great at everything,” Dr Sallam said.

Dr Wu also worked to build connections among his staff outside of the laboratory, hosting barbeques, paintball, and celebrations for the Chinese New Year, said Mirwais Wardak, PhD, principal scientist at Genentech, Inc., in Stanford, California, and a former mentee of Dr Wu.

“Joe also fostered a sense of camaraderie and enjoyment within the lab,” Dr Wardak said. “These occasions allowed us to relax, have fun, and build strong connections beyond the scientific realm.”

June‐Wha Rhee, MD, assistant professor in the Division of Cardiology and the Department of Stem Cell Biology and Regenerative Medicine at City of Hope in Duarte, California, said she remains friends and collaborators with many of her former laboratory mates. She also continues to apply what she learned from Dr Wu.

“Work on something meaningful that has the potential to change the way we treat patients, and do not be afraid of trying new things to reach that goal,” Dr Rhee said.

Dr Zhao said he learned to push himself to be open to new technologies. “Science is moving faster than ever in history, and we will have to learn new technologies to advance our discoveries,” he said.

Dr Wardak and other mentees also said Dr Wu prepared them for the rigors of being a principal investigator, teaching them the importance of resilience and the logistics of running a laboratory, from budgeting to grant writing to contract negotiations.

“Joe equipped me with the essential skills and knowledge I need to navigate the responsibilities of a principal investigator,” Dr Wardak said.

Dr Wu's research and his commitment to mentorship have earned him many honors. He was awarded the Presidential Early Career Award for Scientists & Engineers by President Barack Obama in 2010. He has been elected to the National Academy of Medicine and the National Academy of Inventors and is a fellow of the American Association for the Advancement of Science. He has also been awarded the National Institutes of Health Director's New Innovator Award and Transformative Research Award. In 2022, he was also elected into Academia Sinica of Taiwan, the island nation's equivalent to the National Academy of Sciences, an honor that his parents are most proud of.

With all the work Dr Wu has underway, he has limited free time, but he makes the most of it by spending it with his wife, son, and daughter. He tries to swim daily to stay healthy. On weekends, Dr Wu and his wife volunteer at the church. He enjoys watching documentaries about nature and wildlife. He is also a military history buff with deep respect for the lasting contributions of the nation's military veterans.

“Life can be very hectic for all of us. But when you stop and think, all of us are on this beautiful place called Earth for a very short period of time. So, it is important that we leave this place better than it was when we first came,” Dr Wu said. “As a physician, scientist, and mentor, my contribution is to do translational research that will benefit patients and to train the next generation to carry the baton. It's important for me to pass on my knowledge and to spark their imaginations.”
